# Mechanosensitive Enteric Neurons in the Myenteric Plexus of the Mouse Intestine

**DOI:** 10.1371/journal.pone.0039887

**Published:** 2012-07-02

**Authors:** Gemma Mazzuoli, Michael Schemann

**Affiliations:** Human Biology, Technische Universität München, Freising, Germany; University of California, Los Angeles, United States of America

## Abstract

**Background:**

Within the gut the autonomous enteric nervous system (ENS) is able to sense mechanical stimuli and to trigger gut reflex behaviour. We previously proposed a novel sensory circuit in the ENS which consists of multifunctional rapidly adapting mechanosensitive enteric neurons (RAMEN) in the guinea pig. The aim of this study was to validate this concept by studying its applicability to other species or gut regions.

**Methodology/Principal Findings:**

We deformed myenteric ganglia in the mouse small and large intestine and recorded spike discharge using voltage sensitive dye imaging. We also analysed expression of markers hitherto proposed to label mouse sensory myenteric neurons in the ileum (NF145kD) or colon (calretinin). RAMEN constituted 22% and 15% of myenteric neurons per ganglion in the ileum and colon, respectively. They encoded dynamic rather than sustained deformation. In the colon, 7% of mechanosensitive neurons fired throughout the sustained deformation, a behaviour typical for slowly adapting echanosensitive neurons (SAMEN). RAMEN and SAMEN responded directly to mechanical deformation as their response remained unchanged after synaptic blockade in low Ca^++^/high Mg^++^. Activity levels of RAMEN increased with the degree of ganglion deformation. Recruitment of more RAMEN with stronger stimuli may suggest low and high threshold RAMEN. The majority of RAMEN were cholinergic but most lacked expression of NF145kD or calretinin.

**Conclusions/Significance:**

We showed for the first time that fundamental properties of mechanosensitive enteric neurons, such as firing pattern, encoding of dynamic deformation, cholinergic phenotype and their proportion, are conserved across species and regions. We conclude that RAMEN are important for mechanotransduction in the ENS. They directly encode dynamic changes in force as their firing frequency is proportional to the degree of deformation of the ganglion they reside in. The additional existence of SAMEN in the colon is likely an adaptation to colonic motor patterns which consist of phasic and tonic contractions.

## Introduction

The discovery that the distension induced motor behaviour of the isolated intestine is regulated by the enteric nervous system (ENS) [1]–[4] prompted the search for a mechanosensory neuron in enteric reflex pathways. Very early on, Dogiel suggested that multipolar neurons – referred to as Dogiel type II neurons - may have such a sensory function [5]. Several decades later it was shown in the guinea pig ileum that these neurons – classified electrophysiologically as AH (afterspike hyperpolarisation) neurons [6] indeed respond to sustained circular stretch of the gut wall as well as to mucosal deformation [7], [8]. With these studies the term intrinsic primary afferents neuron (IPAN) was introduced in order to functionally classify AH/Dogiel type II neurons [9]. The IPAN concept has been well received but it is not generally applicable to other species or gut regions. Indeed, the stomach corpus lacks AH/Dogiel type II neurons [10] but exhibits intrinsic reflex behaviour [11]. Moreover, Dogiel type II, AH or AH/Dogiel type II neurons are differently expressed in species other than the guinea pig. Following are just a few examples that question a unique sensory function for these cells. For example, AH neurons are very rare in the human colon [12], AH/Dogiel type II neurons do not respond to sustained distension in muscle paralysed preparations of the guinea pig colon [13], the AH amplitude is not very prominent in the mouse intestine [14], and last but not least, the AH is not a unique property of Dogiel type II enteric neurons in the pig [15].

Recent concepts suggested that mechanosensitivity in the ENS is a property of many different classes of neurons [13], [16], [17]. Recordings with extracellular electrodes revealed early on that mechanical probing of myenteric ganglia activated separate classes of enteric neurons with different firing patterns [18], [19]. In the colon, mechanosensitivity is a feature of so called sensory interneurons, a term that already suggests that these neurons have more than one function. Recently, we identified in the guinea pig ileum rapidly adapting mechanosensensitive enteric neurons (RAMEN) which were activated by mechanical stimuli mimicking deformation during contractile activity [20]. RAMEN fired action potentials in response to von Frey hair probing or intraganglionic micro-volume injections. One of their hallmarks was that they only encoded dynamic deformations while the IPANs [7] and sensory interneurons [13] fired tonically during sustained distension. The response was direct, did not involve synaptic input from other enteric neurons or extrinsic afferents and was independent of muscle tone. Moreover, RAMEN were distinct from previously described mechanosensitive enteric neurons because they received fast EPSPs which strongly suggested that their sensitivity may be modulated. Although most RAMEN were cholinergic they did not specifically express a particular marker. Most strikingly, RAMEN belonged to functionally different classes. Indeed, mechanosensitivity was recorded in interneurons and in even more motor neurons [20]. This prompted us to propose a novel concept on mechanosensitivity in the enteric nervous system which is based on the existence of multifunctional mechanosensitive neurons [17].

Previous work mainly focused on mechanosensitive enteric neurones in the guinea pig. There are only a few studies which addressed the question of whether AH/Dogiel type II neurons behave as mechanosensors in regions outside the ileum or in mice. AH/Dogiel type II neurons were described in the mouse small and large intestine [14], [21]–[23]. Interestingly, these neurons have a neurochemical code that is distinct from those in the guinea pig. In the small intestine these neurons express calcitonin gene-related peptide (CGRP) and the 145-kDa intermediate-molecular-weight neurofilament (NF145kDa) [24] while calretinin (Calret) is a marker for Dogiel type II neurons in the mouse large intestine [25]. There is some evidence that AH/Dogiel type II neurons in the mouse small intestine directly respond to mechanical stimulation by von Frey hair probing and therefore behave like their counterparts in the guinea pig ileum [26].

We argue that the functional relevance of RAMEN and the validity of the concept of multifunctional enteric neurons require that their existence is confirmed in other species and gut regions. The aim of this study was therefore to characterise properties of mechanosensitive myenteric neurons in the mouse ileum and colon. Mice were chosen for the obvious advantage to perform in the future studies in knockout models.

## Methods

### Ethics Statement

All animal procedures are approved by the ethical committee of the government of Oberbayern (Germany) (55.2-1-54-2531.8-210-09) and according to the German guidelines for animal protection and animal welfare.

### Tissue Samples

Male mice C57BI/6 (Charles River, Sulzfeld, Germany) 16 weeks old were killed by cervical dislocation followed by exsanguination. The ileum or the distal colon was quickly removed and further dissected in Carbogen aerated (95% O_2_, 5% CO_2_; pH = 7.40) Krebs solution containing (in mM): 117 NaCl, 4.7 KCl, 1.2 MgCl_2_ 6 H_2_O, 1.2 NaH_2_ PO_4_, 25 NaHCO_3_, 2.5 CaCl_2_ 2 H_2_O, 11 glucose. The mucosa and submucosa were gently removed in order to obtain myenteric plexus preparations still embedded between the two muscle layers. The preparations (5×10 mm) were pinned onto a silicone ring that was placed in a recording chamber continuously perfused with 37°C carbogen bubbled Krebs solution with a rate of perfusion of 11 ml/min. To block synaptic transmission 20 min perfusion of the entire tissue with a low Ca^++^/high Mg^++^ Krebs solution, containing (in mM) 98 NaCl, 4.7 KCl, 16 MgCl_2_ 6 H_2_O, 1.2 NaH_2_ PO_4_, 25 NaHCO_3_, 0.25 CaCl_2_ H_2_O, 11 glucose, was used.

### Ultra-fast Neuroimaging Technique

**Figure 1:**
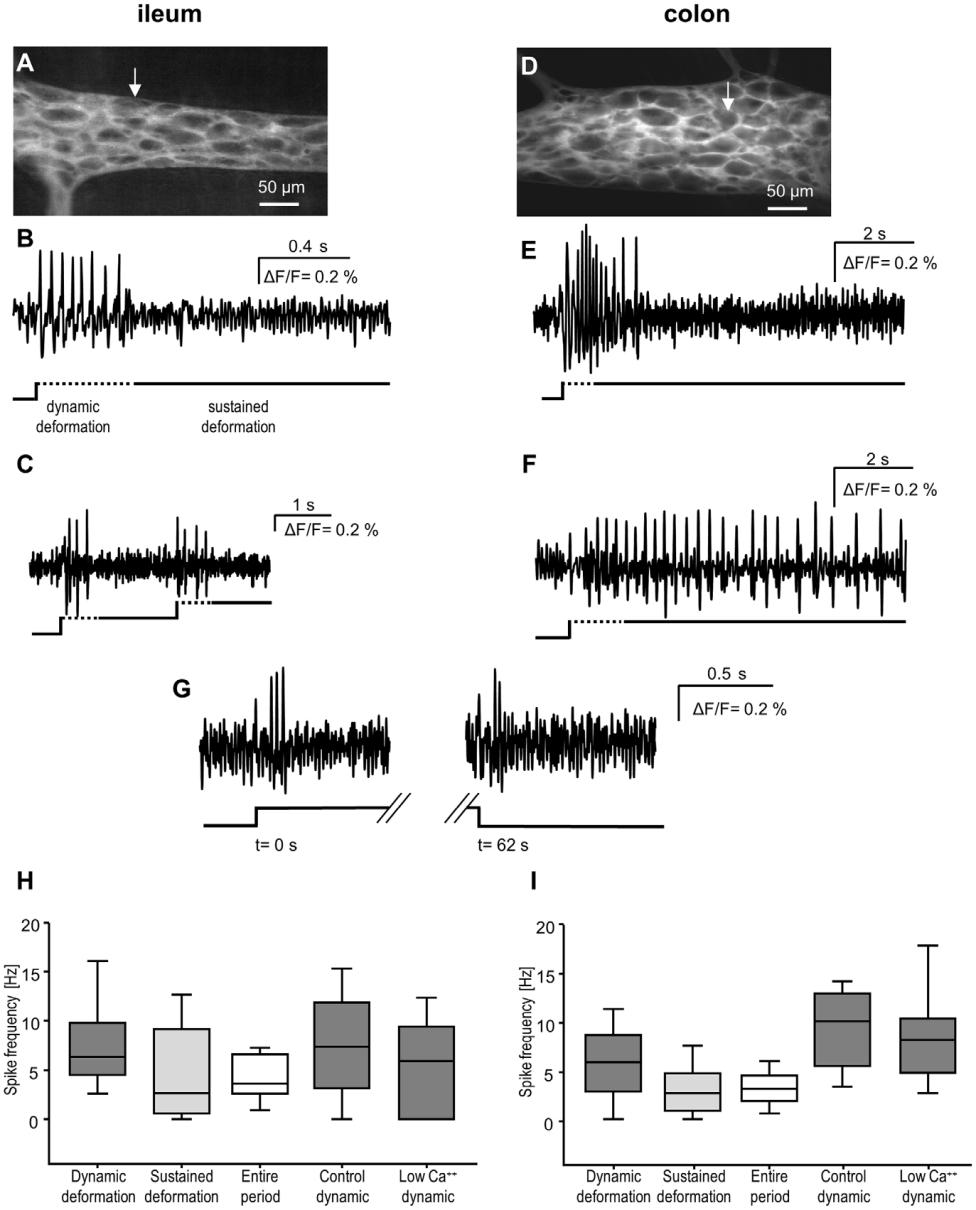
Myenteric neurons in the mouse ileum and colon directly respond to ganglion deformation. Images of Di-8-ANEPPS stained ileal (**A**) and colonic (**D**) myenteric ganglia. **B** and **E** show the responses of the myenteric neurons (marked in A and D by white arrows) from ileum and colon, respectively, to intraganglionic volume injection. The lines below all traces mark the onset of the volume injection (steep upward deflection) and the durations of the dynamic (dotted line) and sustained deformations (solid line). The neurons spiked during the dynamic deformation but stopped firing with the beginning of the sustained deformation. Mechanical deformation induced a rapidly adapting spike discharge, typical for rapidly adapting mechanosensitive enteric neurons (RAMEN). **C** An ileal myenteric neuron responded to two consecutive intraganglionic volume injections with the same spike discharge pattern. **F** Representative trace illustrating the response of a colonic slowly adapting mechanosensitive enteric neuron (SAMEN) that fired throughout the sustained deformation. **G** Trace illustrating responses to applying and releasing a force by advancing and retracting the von Frey hair, respectively. In between the von Frey hair was kept in place for 60 s (disrupted line). Note that the discharge pattern is similar and that spikes occur during dynamic force development. Firing frequency of the myenteric RAMEN in the ileum (**H**) and colon (**I**). RAMEN fired at significantly higher frequency during the dynamic deformation (ileum: ANOVA on Ranks test; p  = 0.009; colon: ANOVA on Ranks test; p  = 0.028). Both in the ileum and colon firing frequency of RAMEN did not change after blockade of synaptic transmission by perfusion of low Ca^++^/high Mg^++^ Krebs solution. All signals have been filtered with a Butterworth filter (low pass 180 Hz, high pass 10 Hz).

An ultra-fast neuroimaging technique to detect signals from a potentiometric dye was used. This technique allowed recording of action potential discharge with a high spatial and temporal resolution as previously described in detail [20]. Briefly, individual ganglia were stained with the fluorescent voltage-sensitive dye Di-8-ANEPPS (1-(3-sulfanatopropyl)-4-[beta[2-(di-*n*-octylamino)-6-naphthyl]vinyl] pyridinium betaine) by local application through a microejection pipette loaded with 20 µM Di-8-ANEPPS dissolved in DMSO and pluronic F-127 containing Krebs solution. The dye staining did not change the electrophysiological properties of the nerve cells [27]. The chamber containing the preparation was mounted onto an epifluorescence Olympus IX 71 microscope (Olympus, Hamburg, Germany) equipped with a 75-W xenon arc lamp (Optosource, Cairn Research Ltd., Faversham, UK). Illumination of the preparation was achieved by a software operated shutter (Uniblitz D122, Vincent Associates, New York, USA). The light emitted by a Xenon lamp excites Di-8-ANEPPS through a modified Cy3 fluorescence filter. This set up allowed us to measure relative changes in the fluorescence (ΔF/F), which is linearly related to changes in the membrane potential [27]. Changes in fluorescence intensity were detected by a CCD camera (80×80 pixels; RedShirt Imaging, Decatur, USA) at a sampling rate of 1 kHz which reliably resolved action potentials. The camera is connected to an inverted microscope (Olympus IX71) and the recordings were made with ×40 or ×100 objectives resulting in a spatial resolution of ∼30 µm^2^ or ∼4 µm^2^ per pixel, respectively. The fluorescent images were acquired and processed by the Neuroplex 8.3 software (RedShirt Imaging). Individual cells can be identified since the dye incorporates into the membrane revealing the outline of individual cell bodies (Fig. 1A, D and Movie S1). The overlay of the signals and the ganglion image allowed the analysis of the responses from single neurons [20].

Bleaching or phototoxicity can be avoided by using several short exposures of 2–4 s. These periods were sufficient to demonstrate the neural responses to mechanical stimuli [20]. During experiments recordings with durations of 1.3–5.0 s yielded reliable and reproducible responses. In some ganglia recordings for up to 10 s were made to detect possible late onset responses.

### Mechanical Stimulation of Ganglia and Neurons

The techniques applied to deform ganglia and ganglion cells were successfully used in our study which identified RAMEN in guinea pig myenteric neurons [20]. These were: the intraganglionic injections of small volumes of Krebs solution and probing with a von Frey hair. We inserted a micropipette into a fibre tract closely to the site where it entered the ganglion. The micropipette was filled with the same oxygenated and buffered Krebs solution that was used for superfusing the preparation. Volumes were injected by a pressure controlled picospritzer (Parker Hannifin Co., Cleveland, OH, USA) for 400 ms. The pressure was adjusted to values that led to deformation of the entire ganglion as visually inspected. As previously described, the intraganglionic volume injection caused two phases of deformation [20]. There was initially a rapid dynamic change in ganglion shape caused by the inflow of the solution. This was followed by a sustained phase of deformation because it took up to 2.5 min for the volume to be redistributed in the ganglionic network (see Movie S1). Unlike in the guinea pig myenteric plexus, where intraganglionic volume injections deformed only one ganglion, the same volume injection deformed up to 10 ganglia in the mouse myenteric plexus. The degree of deformation was then different between ganglia. We used this phenomenon to our advantage and analysed the degree of deformation in different ganglia in the same preparation. As a measure of deformation we calculated the changes in the surface area of a ganglion before intraganglionic injection and after injection at the time when the ganglion was maximally deformed. In addition, we measured the duration between the start and end of the dynamic deformation.

The second stimulus was probing of the ganglia with an 8 µm diameter carbon fibre (exerting a force of 62±4 µN) which was connected to a motorized micromanipulator (DC-3K, Märzhauser, Wetzlar, Germany) to control advancement of the hair. The hair was placed 1 to 2 µm above the ganglion and then advanced to create ganglion deformation with a step size of 10 µm, then left in place for 1 min and finally retracted with a 10 µm step. In preliminary experiments mechanical stimulations were repeated at least three times at 5–10 min intervals to ensure that there was a genuine response rather than cell damage induced spike discharge. In addition, after the experiments, any cell damage could be excluded by immunohistochemical staining that allowed us to verify intact cell morphology.

### Immunohistochemistry

In order to study the neurochemical code of the mechanosensitive neurons immunohistochemistry was performed. Tissue specimens were fixed 4 h at room temperature in a solution containing 4% paraformaldehyde and 0.2% picric acid in 0.1 mol/l phosphate buffer and then washed (3×10 min) in phosphate buffer. Whole mount preparations were first incubated in phosphate buffered saline (PBS)/NaN_3_ (0.1%)/horse serum (HS, 4%) for 1 h at room temperature followed by 24 h and 3 h incubation with the primary and secondary antibody, respectively. As primary antibodies we used goat anti-choline acetyltransferase (ChAT; 1∶200; AB144P; Chemicon), rabbit anti-Neurofilament 145 kDa (NF 145 kDa; 1∶500; AB1987; Millipore, Schwalbach/Ts, Germany), rabbit anti-nitric oxide synthase (NOS; 1∶200; 210501R025; Alexis-Enzo Life Sciences GmbH, Lörrach, Germany) and goat anti-calretinin (Calret; 1∶3000; CG1; Swant, Switzerland). As secondary antibodies we used donkey anti-rabbit conjugated to Carbocyanin (CY2; 1∶500; 711225152; Dianova, Hamburg, Germany), donkey anti-goat conjugated to Carbocyanin (CY5; 1∶50; 705225147; Dianova) or donkey anti-rabbit conjugated with biotin (1∶50; 711065152; Dianova) and then streptavidin AMCA (1∶200; 016150084; Dianova). Finally, specimens were washed in PBS, mounted on poly-l-lysine-coated slides and cover slipped with a solution of PBS (pH 7.0)/NaN_3_ (0.1) containing 65% glycerol. The preparations were examined with an epifluorescence microscope (Olympus), equipped with appropriate filter blocks. Pictures were acquired with a video camera connected to a computer and controlled by Scion image software (Scion Corp., Frederick, MD, USA). Frame integration and contrast enhancement were employed for image processing.

### Data Analysis and Statistic

We counted the number of dye-labeled neurons in the ganglia and analysed the number of mechanosensitive neurons per ganglion and the frequency of action potentials discharge. In addition, spike discharge frequency during the dynamic deformation was separately analysed. These values were then correlated with the degree of ganglion deformation. The duration of spike discharge and the occurrence of the last action potential were calculated. We additionally analysed the instantaneous frequency (reciprocal of the interval between sequential action potentials) and averaged the values for dynamic and sustained deformation as well as for the entire period.

For signal and image analysis we used Neuroplex 8.3.2 (RedShirt Imaging), Igor Pro 6.03 (Wavemetrics Inc., Lake Oswego, OR, USA) and Image J 1.43u (Wayne Rasband, National Institute of Health, USA) software. For the movie editing the program Adobe Premiere Pro (Adobe Systems Inc. San Jose, CA, USA) was used. The statistical analyses and graphics were performed with Sigmaplot 12.0 (Systat Software Inc., Erkrath, Germany). All data are presented as mean ± standard deviation or, when not normally distributed, as median values together with the 25% and 75% quartiles given in brackets. Differences in the spike frequency between several control stimulations were performed with a one way repeated measures analysis of variance (ANOVA) test. Action potential frequency fired from the same neuron after 2 subsequent stimuli were tested with a paired t-test. Correlation analyses were performed with Pearson Product Moment Correlation. Comparisons between data from the small and large intestine were made with Mann-Whitney Rank Sum Test. To test the differences in action potential frequency before and after blockade of synaptic transmission we used repeated measures ANOVA on ranks. Differences and correlations were considered significant when P was <0.05.

## Results

### Mechanosensitive Myenteric Neurons in the Mouse Ileum

In the ileum mechanical stimulation by intraganglionic volume injection was performed in 51 ganglia from 12 mice. The intraganglionic injection resulted in an initial dynamic deformation of the ganglia which lasted 306 [153/401] ms, followed by a sustained deformation which lasted for 150±40 s; during this time the volume was redistributed in the ganglionic network and the ganglion regained its original shape (see Movie S1).

The mean number of neuronal cell bodies per ganglion was 19±6. Ganglion deformation evoked a spike discharge in 22±11% neurons per ganglion (range 5–55%). Of these mechanosensitive neurons 20% (47 neurons) fired one action potential only. All other 188 mechanosensitive neurons fired at least 2 and up to 59 spikes (see example trace in Fig. 1B). During the dynamic deformation, the spike discharge rate was 6.3 Hz. The spike discharge ceased during the sustained deformation which resulted in an overall spike discharge rate of 3.6 Hz (Fig. 1H). The instantaneous frequency during dynamic deformation was 18.8 [10.0/32.3] Hz; during sustained deformation 5.8 [0.8/15.8] Hz and for the entire period it was 13.1 [7.0/18.9] Hz.

The rapid adaptation was not the result of desensitization to the deformation. Deforming the ganglion twice within a 2 s interval revealed identical numbers of mechanosensitive neurons (both 22±9%, 5 ganglia from 3 mice) which fired during the dynamic deformation with at comparable frequencies (5 [5/5] Hz versus 3 [1.7/8.7] Hz) (Fig. 1C).

**Figure 2:**
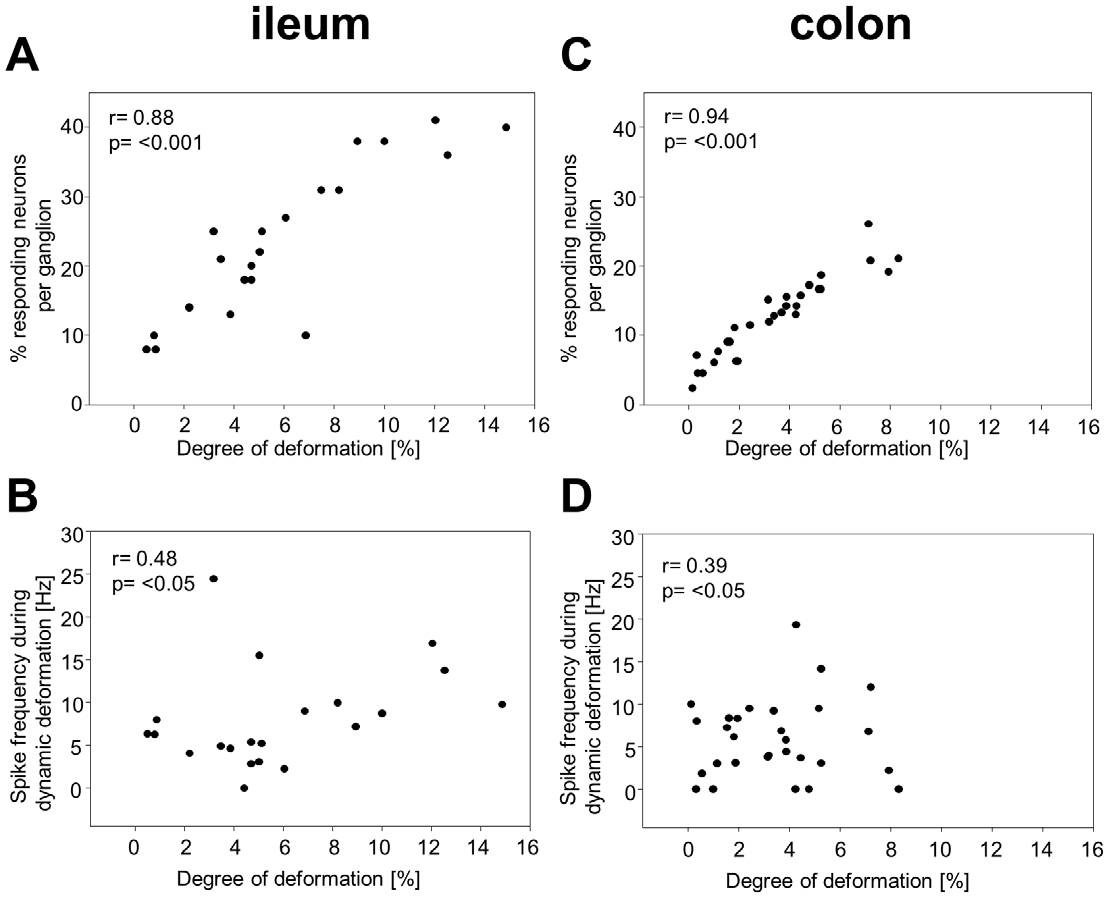
The proportion of neurons responding to ganglion deformation as well as their spike frequency is significantly correlated to the degree of ganglion deformation. Ganglion deformation was assessed by % change in the ganglionic area in the ileum and colon. The correlation analysis was performed with Pearson Product Moment Correlation (P<0.05; R  =  correlation coefficient).

The degree of deformation expressed as percentage change in the ganglionic area after intraganglionic volume injection was 5.1 [3.8/8.9] % (23 ganglia from 8 mice). Of note, the percentage of mechanosensitive neurons as well as the spike frequency during the dynamic deformation positively correlated (p<0.05) with the degree of ganglion deformation (Fig. 2A, B).

Once started, the spike discharge lasted on average 239 [100/402] ms. The time of occurrence of the last action potential during burst firing was 389 [243/686] ms after the beginning of the dynamic deformation. To check for possible late onset responses we used longer recording periods in 4 ganglia and found that none of the responding neurons fired action potentials beyond 2 s after the stimulus.

The spike discharge pattern was rapidly adapting (see Fig. 1B, C) and very similar to that of mechanosensitive myenteric neurons in the guinea pig. Therefore mechanosensitive myenteric neurons in the mouse with this firing behaviour were also termed RAMEN.

In order to study the reproducibility of the responses we performed experiments (5 ganglia from 4 mice) with 3 mechanical stimulations 10–15 min apart. These stimulations provoked each time a similar change in ganglionic area which varied between the stimuli less than 0.21%. In these experiments we found a high reproducibility of the responses: the number of mechanosensitive neurons per ganglion (20±9%, 20±8% and 19±8%, respectively) and the overall spike discharge (2.4 [1.5/4.3] Hz, 2.4 [1.4/4.6] Hz and 2.3 [1.6/4.0] Hz, respectively) remained stable.

### Mechanosensitive Myenteric Neurons in the Mouse Distal Colon

In the distal colon mechanical stimulation was performed in 91 myenteric ganglia from 14 mice. Like for the ganglia in the ileum the intraganglionic volume injection resulted in a two phase’s deformation. An initial dynamic deformation of the ganglia which lasted for 320 [248/433] ms was followed by a sustained deformation which lasted for 175±35 s.

The mean number of neuronal cell bodies per ganglion was 22±10. Of these, 14±9% neurons per ganglion responded to the mechanical stimulation (range 2–26%) (Fig. 1D-F). About 20% (46 neurons) of the mechanosensitive neurons fired one action potential only. All the other 184 mechanosensitive neurons fired 2–29 action potentials. The spike frequency during the dynamic deformation was 5.8 Hz and declined during the sustained deformation which resulted in an overall spike discharge 3.1 Hz (Fig. 1I). The instantaneous frequency was 9.4 [0.7/21.0] Hz during dynamic deformation, 5.4 [0.4/12.9] Hz during sustained deformation and 8.7 [3.9/12.9] Hz for the entire period.

Like RAMEN in the ileum, two subsequent stimuli within an interval of 2 s activated the same mechanosensitive neurons in the colon (both 11±7% per ganglion, 6 ganglia from 2 mice) without significant changes in the firing rate during the dynamic deformation (5 [2.5/10] Hz versus 7.5 [5/12] Hz).

The degree of deformation calculated as percentage change in the ganglionic area was 3.4 [1.6/5.0] % (29 ganglia from 6 mice) and significantly smaller than in the ileum. Like in the ileum the percentage of mechanosensitive neurons as well as the firing frequency during the dynamic deformation was positively correlated (p<0.05, Pearson Product Moment Correlation) with the degree of ganglion deformation (Fig 2C, D). In two ganglia it unintentionally happened that two consecutive intraganglionic volume injections (15 min apart) produced a vastly different ganglion deformation. In the first ganglion the two deformation stimuli induced a change in ganglionic area of 4.2% and 16.7%, respectively. In this ganglion the first stimulus activated one neuron and the second activated 4 neurons. The same phenomenon was observed in the second ganglion where an increase of deformation from 1.9% to 5.2% increased the number of mechanosensitive neurons from 1 to 3 neurons. Although we could not systematically study this behaviour, the observation suggested the existence of low and high threshold RAMEN.

Once started, the spike discharge lasted on average 540 [154/1,196] ms. The last spike occurred 1,067 [409/1,482] ms after the beginning of the dynamic deformation. The majority of mechanosensitive neurons (127 out of 159) showed a rapidly adapting spike discharge typical for RAMEN (Fig. 1E). The remaining 32 mechanosensitive neurons showed on-going spike discharge also during the sustained deformation. About 7.1% of all mechanosensitive neurons in the colon (12 out of 32 neurons) even fired throughout a 10 s recording period at 4.3 [2.8/8.3] Hz (Fig. 1F). However, also these neurons fired at a significantly higher rate during the dynamic deformation (8.3 [6.3/11.9] Hz; p = 0.02). This kind of response would be considered as slowly adapting, thus we named these neurons slowly adapting mechanosensitive enteric neurons (SAMEN).

### Experiments with Von Frey Hair Probing

Like their counterparts in the guinea pig myenteric plexus, RAMEN in the mouse myenteric plexus respond to von Frey hair probing with spike discharge during dynamic deformation. As already described in our study in guinea pig myenteric plexus, intraganglionic volume injection was much superior to von Frey hair stimulation regarding reliability and reproducibility of the response. This was mainly due to the inability to repeatedly position the hair exactly on the same spot. The disadvantage of the deformation evoked by volume injection was the slow recovery. Therefore, onset and rapid offset of the stimulus can only be controlled with von Frey hair probing. We used von Frey hair probing to specifically address the question whether RAMEN respond to loading (onset of deformation by advancing the hair) as well as to unloading (offset of the stimulus by retracting the hair). Such experiments were performed in 20 mechanosensitive neurons (17 ganglia from 3 mice). The spike frequency of RAMEN was identical between loading and unloading (1.4 [0.7/2.8] versus 1.4 [0.5/1.8] Hz) (Fig. 1G).

We performed experiments (4 mice, 12 ganglia) where we used in the same ganglia the von Frey hair probing and the intraganglionic volume injection. With the von Frey hair one deforms a relatively small area of the ganglion while distant areas of the ganglion showed no noticeable deformation (see also [20]).With the intraganglionic volume injection we could achieve deformations of the entire ganglion. As expected, deformation by intraganglionic volume injection activated more mechanosensitive neurons than von Frey hair probing (16.3±10% versus 7.8±5%; p = 0.016). The overall action potential frequency was also significantly larger (p = 0.001) with intraganglionic volume injection (2.8 [2.2/6.1] Hz versus 1.1 [0.5/1.6] Hz) as was the instantaneous frequency (11.1 [5.4/17] Hz versus 3.2 [1.5/8.0] Hz). However, most importantly, neurons that responded to von Frey hair probing always responded to intraganglionic injection. The lower responsiveness of RAMEN to von Frey hair probing may be due to the faster and shorter dynamic deformation.

### Mechanosensitive Neurons Respond to Mechanical Stimulation after Blockade of Synaptic Transmission

The blockade of synaptic transmission with low Ca^++^/high Mg^++^ did not change in the ileum the number of mechanosensitive neurons responding to ganglion deformation (17±11% of the versus 17±10%, 8 ganglia from 4 mice). There was also no change in the overall spike discharge rate (3.5 [1.5/6.2] Hz versus 2.9 [1.5/4.9] Hz) or in action potential frequency during the dynamic deformation (7.7 Hz versus 6.7 Hz) (Fig. 1H).

Likewise, in the colon the percentage of mechanosensitive neurons per ganglion remained unchanged (10±3% in both cases, 6 ganglia from 3 mice). Moreover, the action potential frequency during the dynamic deformation 10.0 Hz versus 8.1 Hz (Fig. 1I) and the overall spike frequency were not significantly changed (1.3 [0.8/4.4] Hz versus 1.6 [0.9/4.7] Hz). In these experiments 2 SAMEN fired throughout the sustained deformation at 11.8 and 4.0 Hz their spike discharge was not changed in low Ca^++^/high Mg^++^ (9.4 and 5.9 Hz).

Of note, the percentage of mechanosensitive neurons as well as the firing frequency during the dynamic deformation was still positively correlated (p<0.05) with the degree of ganglion deformation during synaptic blockade by low Ca^++^/high Mg^++^.

### Responses of Mechanosensitive Responses were not Affected Lowering Intraganglionic K^+^ Concentration

We assumed that the intra- and extraganglionic K^+^ concentrations are in equilibrium during continuous Krebs perfusion. We nevertheless performed experiments with intraganglionic injection of low K^+^ (1 mM) Krebs solution in order to account for the possible buffering capacity of glia cells. Intraganglionic injection of low K^+^ solution (12 colonic ganglia in preparations from 4 mice) activated 16±9% neurons per ganglion which fired at 7.5 [3.2/9.8] Hz during the dynamic and at 2.8 [2.0/5.8] Hz during the sustained deformation. Both the percentages of mechanosensitive neurons as well as their firing frequencies were comparable to injection of Krebs solution containing 4.7 mM K^+^ (see data above).

### Neurochemical Coding and Size of Mice RAMEN

**Figure 3:**
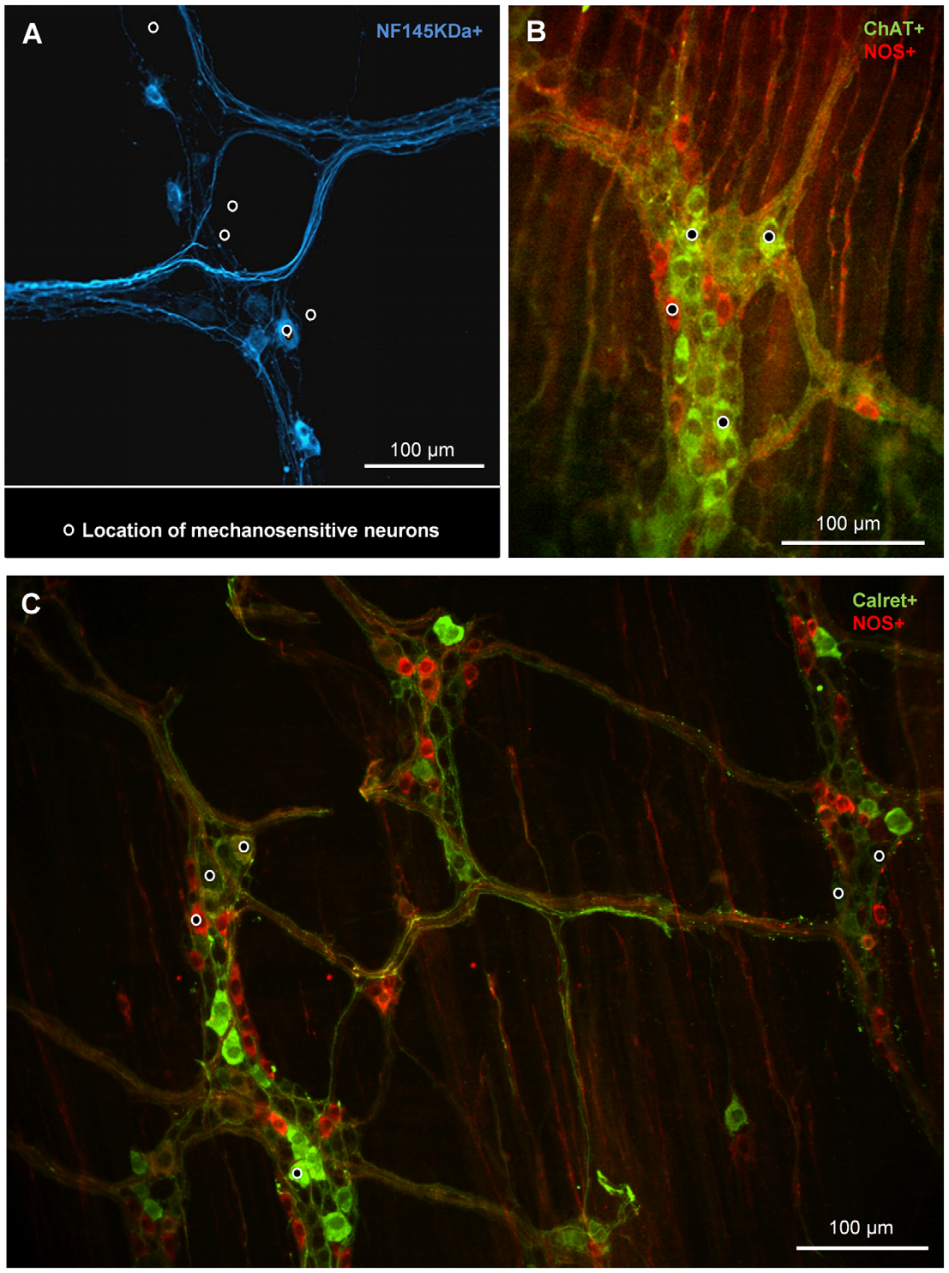
Most mechanosensitive enteric neurons are cholinergic but did not express the proposed markers for sensory enteric neurons (NF145KDa in the ileum; calretinin in the colon). In all panels the location of mechanosensitive neurons in the ganglion is marked by black dots with white circles. **A** NF145KDa immunoreactive (IR) neurons in the ileum, 5 mechanosensitive neurons are marked, only 1 is NF145KDa positive. **B** ChAT and NOS immunoreactive neurons in the ileum, 4 mechanosensitive neurons are marked, 3 are ChAT- and 1 is NOS-IR. **C** NOS and Calret-IR neurons in the colon. 6 mechanosensitive neurons are marked, 3 are negative for both markers, one is positive for both, 1 is NOS- and 1 is Calret-IR.

Based on previous studies, the putative mechanosensitive myenteric neurons in the mouse (AH/Dogiel type II neurons) can be identified by NF145kDa-IR in the ileum [24] and Calret-IR in the distal colon [25]. We used these two markers to test if the RAMEN population overlaps with the previously identified mechanosensitive neuronal populations. In the ileum (18 ganglia from 5 mice) only 10% of RAMEN (7 out of 70) were NF145kDa-IR (Fig. 3A). In the distal colon only 28% of mechanosensitive neurons (19 out of 69) were Calret-IR (24 ganglia, 3 mice) (Fig. 3C).

Additional markers where used to identify the cholinergic or nitrergic phenotype of RAMEN. In the ileum (16 ganglia from 2 mice) double staining revealed that 79% of mechanosensitive neurons (62 out of 78 ) were ChAT-IR, 12% (9 out of 78) were NOS-IR and the remaining 9% (7 out of 78) were negative for both markers (Fig. 3B). ChAT-IR in the distal colon is not reported because the staining was not strong enough to reliably reveal neuronal somata. NOS-IR was found in 23% (16 out of 69) of mechanosensitive neurons in the distal colon (Fig. 3C).

Small neurons may be easier to activate because of their lower input resistance; but different cell sizes did not affect the identification of RAMEN. There was no difference in cells size between mechanosensitive neurons (n = 15; 26.3±1.6 µm and 16.1±1.0 µm long and short axis, respectively) and those that did not respond to ganglionic deformation (n = 15; 25.1±0.8 µm and 14.1±1.2 µm).

### Comparison between RAMEN in Mouse Ileum and Distal Colon

The behaviour of most mechanosensitive neurons in the ileum and distal colon was comparable and characterised by rapidly adapting spike discharge. Therefore, the use of the term RAMEN seems appropriate. The number of neurons per ganglion was comparable in the ileum (19±6) and distal colon (22±10), despite the finding that myenteric ganglia in the ileum were significantly smaller than those in the colon (34,206 [19,032/38,872] µm^2^ versus 61,232 [47,903/76,520] µm^2^). This may explain why the degree of deformation was on average significantly higher in the ileal myenteric plexus (p = 0.005). Nevertheless, the duration of the dynamic deformation was similar in both regions. Although the overall spike frequency as well as the firing frequency during the dynamic deformation did not significantly differ between the two regions, colonic RAMEN fired for a longer period than RAMEN in the ileum (p = 0.003).

The percentage of mechanosensitive neurons appeared to be significantly larger in the ileum (22±11%) than in the in the colon (14±9%). This is likely an experimental bias as the degree of ganglion deformation was in general smaller in the colon. Analysis of only those ganglia that were deformed to a similar degree (variation of ≤0.3% in ganglion area) revealed that neither the percentage of responding neurons nor the firing frequency during dynamic deformation was significantly different between the two regions: 19±8% responding neurons in the ileum fired at 5.8 [4.2/9.9] Hz and 14±6% responding neurons fired at 8.8 [6.9/9.6] Hz in the colon.

## Discussion

This study identified in the mouse small and large intestine mechanosensitive neurons with characteristics and properties comparable to previously identified RAMEN in the guinea pig ileum [20]. To the best of our knowledge, this represents the first report that mechanosensitive enteric neurons have almost identical properties across species and regions. There is no significant difference in the proportion of RAMEN (p = 0.146) or in their spiking frequency (p = 0.612) between guinea pig and mouse myenteric ganglia.

By analogy to the guinea pig, mouse RAMEN fired action potentials during the dynamic deformation. The response declined with the decay in deformation (rapid adaptation) and ceased when the ganglion entered the phase of sustained deformation. This behaviour is not due to desensitization because spike discharge occurred in response to two consecutive mechanical stimuli applied within 2 seconds. This pattern of response is likely to reflect the type of muscle activity present in the small intestine. Phasic contractions are in fact dominant in the ileum [28]. In the distal colon, where phasic and tonic muscle activity occurs [28], [29], we expected to see mechanosensitive neurons with different response patterns. In this region we indeed identified SAMEN – the slowly adapting mechanosensitive enteric neurons. These neurons fired action potentials during the dynamic and throughout the sustained deformation. This property makes them candidates for encoding long lasting deformation during sustained colonic muscle tone.

Several results extended our previous findings in the guinea pig ileum. Firstly, RAMEN fired during the onset of ganglionic deformation by advancing the von Frey hair but also when releasing the force by retracing the hair. This suggested that they are able to encode dynamic deformations independent of whether we apply or release the force. Secondly, the proportion of RAMEN responding to deformation and their firing frequency strongly correlated with the degree of ganglionic deformation. Based on the result that there is still a positive correlation between firing rate of RAMEN and ganglion deformation after synaptic blockade, we conclude that sensitivity to deformation is not influenced by neural tone but rather an intrinsic property of RAMEN. Thirdly, we demonstrated that firing rate of mechanosensitive enteric neurons not only relates to the stimulus strength but, even more important, to the degree of ganglion deformation as a result of varying stimulus strengths. Our results are in line with previous suggestion that guinea pig enteric neurons respond to ganglion deformation that occurs during contraction and relaxation of the gut [20]. Positive correlation between stimulus strength and firing rate of enteric mechanosensitive neurons has been reported before [7], [19], [20]. Moreover, AH/Dogiel type II neurons as well as sensory interneurons fire in response to spontaneous contractions [7], [30].

Markers for putative sensory Dogiel Type II neurons in the mouse ileum (NK145KDa [24]) and colon (Calret [25]) were not commonly expressed in RAMEN. This agrees with our findings in the guinea pig ileum where Calbindin as a marker for AH/Dogiel type II neurons was rarely present in RAMEN and supported our previous conclusion that RAMEN differ from the previously described IPANs. Generally, these findings let us assume that non-AH neurons also have mechanosensitive functions, a conclusion that agrees with previous findings in the guinea pig [13], [20]. It is of note that the proportion of cholinergic RAMEN is almost identical in the mouse (79%) and guinea pig (72%) ileal myenteric plexus.

In summary, our findings support the existence and functional relevance of RAMEN across species and regions. Basic properties of RAMEN, such as firing pattern in response to ganglion deformation, their proportion and cholinergic phenotype are almost identical in mouse and guinea pig. This suggests that RAMEN are fundamental for mechanosensitivity in the ENS.

## Supporting Information

Movie S1 **Activation of enteric mechanosensitive neurons by ganglionic deformation.** Slow motion movie of the ganglionic deformation during intraganglionic volume injection (original length 1.3 s). The outlines of the neurons are visible because the voltage sensitive dye Di-8-ANEPPS incorporates into the outer membrane. The movie shows the deformation during the volume injection and at the same time the colour coded action potential discharge (red flashes). Locations of the 2 neurons that fire action potentials in response to intraganglionic volume injection are indicated by red arrows. Note that with this magnification activity in one neuron is detected by several pixels. The two traces to the left show the responses of the two mechanosensitive neurons. Each action potential corresponds to the colour coded activity (red flashes). In the upper part of the movie onsets of the dynamic and sustained deformations are indicated.
(AVI)
